# Association between accelerated biological aging and colorectal cancer: a cross-sectional study

**DOI:** 10.3389/fmed.2025.1533507

**Published:** 2025-02-21

**Authors:** Sai Wang, Keyu Wang, Xiu Wang

**Affiliations:** ^1^Department of Thoracic Surgery, Jining Third People’s Hospital (Yanzhou District People’s Hospital of Jining City), Jining, China; ^2^Department of Hepatobiliary Surgery, Jining Third People’s Hospital (Yanzhou District People’s Hospital of Jining City), Jining, China; ^3^Jiangsu Province (Suqian) Hospital, Suqian, China

**Keywords:** biological age, KDMAge, PhenoAge, colorectal cancer, elderly

## Abstract

**Background:**

Biological age (BA) is regarded as a more accurate marker of aging than chronological age and is commonly used to assess associations with age-related diseases. The relationship between BA measures and the colorectal cancer (CRC) has not yet been investigated.

**Methods:**

This study utilized data from the National Health and Nutrition Examination Survey. BA was quantified using the Klemera–Doubal method age (KDMAge) and phenotypic age (PhenoAge), based on 13 common clinical biomarkers. The prevalence of CRC across quartiles of BA indicators was compared using weighted Chi-square tests. Weighted multivariable logistic regression models were used to assess the association between BA indicators and CRC.

**Results:**

A total of 36,684 participants were included. The weighted prevalence of CRC showed a significant and consistent upward trend across ascending quartiles of chronological age, KDMAge, and PhenoAge, even within gender and age subgroups (all *P* for trend < 0.05). In the total population and gender subgroups, higher quartiles of PhenoAge acceleration showed a higher weighted prevalence of CRC compared to lower quartiles (*P* for trend < 0.05). Accelerated PhenoAge was significantly associated with a higher prevalence of CRC (OR = 1.767, 95% CI: 1.236–2.524, *P* = 0.002). However, accelerated PhenoAge was associated with the increased prevalence of CRC only in individuals older than 65 years (OR = 1.655, 95% CI: 1.143–2.397, *P* = 0.008).

**Conclusion:**

Biological aging are positively associated with the prevalence of CRC regardless of gender, particularly among the elderly.

## 1 Introduction

With the acceleration of population aging, the incidence of diseases associated with older adults is rising sharply, posing significant challenges to families and the socio-economic landscape. Cancer is a class of malignancies closely associated with age, with over 70% of cancer patients being over the age of 65 (1). Colorectal cancer (CRC) is one of the most common malignancies worldwide (2). According to GLOBOCAN 2020 estimates, there were approximately 1.93 million new cases of CRC globally in 2020, with about 930,000 deaths, ranking it third in incidence and second in mortality among all cancers (3). Colon cancer, a significant type of CRC, often presents with atypical early symptoms. Early screening and prevention of CRC continue to face numerous challenges (4–6).

In recent years, advancements in CRC screening programs and improvements in treatment modalities have led to a steady decline in the incidence of CRC in developed countries (2). However, among the elderly, the incidence and mortality rates of CRC remain alarmingly high. In 2020, approximately 54% of new CRC diagnoses in the United States were in patients aged over 65 years, significantly higher than the 12% among those under 49 years and 34% between 50 and 64 years. Additionally, about 68% of CRC deaths occurred in patients aged over 65 years (7). Previous research has found that the prognosis of CRC is closely related to the timing of diagnosis and treatment; early-stage CRC has a 5-year survival rate exceeding 90%, whereas advanced-stage CRC has a survival rate of less than 5% (8). Therefore, early identification of high-risk groups among the elderly is particularly crucial in reducing the significant health economic burden on national and societal levels.

Given the close relationship between the incidence and mortality rates of CRC and age, identifying high-risk groups for CRC during the aging process may be an effective strategy. Aging is a complex biological process driven by the accumulation of long-term damage at the molecular and cellular levels (9). These physiological changes can accelerate the decline in body functions. Thus, the onset and progression of various diseases are influenced to varying degrees by the aging of body tissues and organs (10, 11). Previous studies have indicated that chronological age can only serve as a retrospective measurement method (11) and does not fully reflect an individual’s true state of aging, nor can it identify variations in the real aging state among individuals of the same chronological age. With the continuous development of biological detection technologies, markers reflecting biological aging have become more abundant, breaking the limited perception that aging is synonymous with growing older and significantly enhancing our understanding of biological aging.

Biological age (BA), involving the aging of multiple body biological systems (12), is a primary risk factor for most age-related diseases, physical and cognitive impairments, and death (13). Increasing evidence suggests that BA is closely related to age-related diseases. For instance, promising applications include a series of algorithms applied to DNA methylation data, which can estimate a person’s BA or risk of death (14, 15), although these methods are not readily implemented clinically. Another promising BA is derived from algorithms based on blood chemistry and other clinical data, namely the Klemera–Doubal method age (KDMAge) and phenotypic age (PhenoAge) (16, 17). KDMAge is calculated using clinical biomarkers like blood pressure, albumin levels, creatinine, and total cholesterol, along with other health markers. PhenoAge uses a set of nine clinical biomarkers, including factors like albumin, alkaline phosphatase, glucose, and C-reactive protein. KDMAge emphasizes a more linear relationship between biological and chronological age, while PhenoAge models aging in a manner more directly associated with mortality risk, considering multiple biomarkers (16, 17). Accelerated KDMAge or PhenoAge refers to the difference between a person’s estimated biological age and their chronological age. Compared to CA, BA calculated based on biochemical and functional indicators from healthy populations can more accurately reflect an individual’s physiological condition and the risk of contracting age-related diseases and death (16). Additionally, compared to chronological age, using BA can help health professionals identify high-risk individuals in a timely manner, potentially preventing disease onset (18).

To date, the association between BA measures and CRC has not been investigated. To address this gap, this study aims to assess the relationship between KDMAge and PhenoAge and the prevalence of CRC, and to further validate this relationship across different population subgroups.

## 2 Materials and methods

### 2.1 Study design and population

This study used data from the National Health and Nutrition Examination Survey (NHANES), a cross-sectional survey conducted by the National Center for Health Statistics (NCHS) under the Centers for Disease Control and Prevention. Ethical approval for this study was granted by the NCHS Ethics Review Board (NHANES 1999–2004: Protocol #98-12; NHANES 2005–2010: Protocol #2005-06; NHANES 2011–2018: Protocol #2011-17, #2018-01), and all NHANES participants provided written informed consent. Data and detailed information about NHANES can be accessed from https://www.cdc.gov/nchs/nhanes/.

**FIGURE 1:**
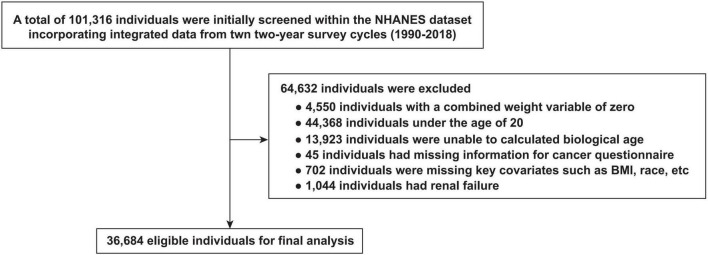
Flow chart of participants selection from the NHANES 1999–2018.

We integrated data from ten biennial NHANES surveys conducted between 1999 and 2018, encompassing a total of 101,316 individuals. According to the exclusion criteria, 64,632 individuals were removed due to weight of zero, under the age of 20, unable to have their biological age calculated, missing information related to cancer questionnaires, missing key covariates, and had renal failure. Finally, 36,684 eligible individuals were included in the final analysis. The details of exclusion criteria and population screening process are presented in Figure 1.

### 2.2 Biological aging assessment

The KDMAge and PhenoAge measurements quantify systemic integrity deficits associated with aging by combining information from various clinical biomarkers at the individual level (19). The KDMAge corresponds to an individual’s predicted BA, reflecting their physiological function compared to a reference population. The KDM BA algorithm is derived from a series of regressions of individual biomarkers on chronological age within the reference population. The KDMAge calculation involves clinical indicators such as systolic blood pressure, albumin, alkaline phosphatase, blood urea nitrogen, creatinine, C-reactive protein, glycated hemoglobin, and total cholesterol levels. PhenoAge represents the predicted age corresponding to an individual’s mortality risk, derived from a multivariable analysis of mortality hazards in a reference population. The PhenoAge algorithm utilizes clinical markers including albumin, alkaline phosphatase, creatinine, glucose, C-reactive protein levels, lymphocyte percentage, mean cell volume, red cell distribution width, and white blood cell count. To evaluate accelerated biological aging, residuals of BA were computed by regressing the BA measures on CA at the time of biomarker measurement, defined as KDMAge acceleration and PhenoAge acceleration values. Acceleration values exceeding zero indicated accelerated aging for KDMAge or PhenoAge, whereas values at or below zero indicated non-accelerated aging for these measures.

### 2.3 CRC diagnosis

Colorectal cancer diagnosis was identified based on self-reported previous diagnoses using questionnaire items MCQ220 and MCQ230A-D from the Medical Conditions section of the Questionnaire Data. MCQ220 captures responses to the question, “Ever told you had cancer or malignancy?” as posed by a doctor or other health professional. MCQ230A-D records up to four types of cancer in response to the question, “What kind of cancer?” Responses including the “Colorectal” were considered indicative of a CRC diagnosis.

### 2.4 Covariates

A comprehensive set of covariates was considered to potentially confound the relationship between BA measures and CRC. These covariates included age, gender, race, body mass index (BMI), history of diabetes, history of hypertension, smoking status, and alcohol consumption. The racial categories included “Mexican American,” “Other Hispanic,” “Non-Hispanic White,” “Non-Hispanic Black,” and “Other Race – Including Multi-Racial.” BMI was calculated by dividing weight (kg) by the square of height (m). Histories of diabetes and hypertension were identified based on self-reported diagnoses. Smoking status was categorized as “Not at all,” “Some days,” or “Every day,” with the “Every day” category defined as current smokers. The individual who having at least 12 alcoholic drinks in the previous year was defined as a drinker.

### 2.5 Statistical analysis

Due to NHANES’ complex multistage probability sampling design, 10 cycle weights were calculated and applied in all analyses to provide nationally representative estimates. Continuous variables were presented as medians with interquartile ranges and compared using the weighted Wilcoxon rank-sum test, while categorical variables were described by weighted percentages and compared using the weighted Chi-squared test. Weighted linear regression models were used to examine the linear trends for CRC prevalence across increasing quartiles of BA measures. After adjusting for covariates, the associations between BA measures and the prevalence of CRC were assessed using weighted multiple logistic regression models. Results were presented as odds ratios (ORs) with 95% confidence intervals (CIs) and *P* values. The analyses were repeated in subgroups stratified by gender (male and female) and age (<65 years and ≥65 years). All BA measures were standardized. A two-sided *P* < 0.05 was considered statistically significant. All statistical analyses were conducted using R software version 4.3.1.

## 3 Results

### 3.1 Basic characteristics of the study population

The weighted basic information table is provided in Supplementary Table 1. Table 1 summarizes the basic characteristics of the study population, divided into two age groups: under 65 years and 65 years and over. The total sample size was 36,684, with 27,841 participants under the age of 65 and 8,843 participants aged 65 and over. Statistical results indicate significant differences between the groups in age, racial composition, BMI, liver function markers (ALT and AST), history of hypertension and diabetes, and smoking and drinking habits (all *P* < 0.001).

**TABLE 1:** Basic characteristics of the study population.

	Overall	Age < 65 years	Age ≥ 65 years	*P*-value
*N*	36,684	27,841	8,843	
Age (years)	48.00 (34.00∼64.00)	41.00 (30.00∼53.00)	74.00 (69.00∼80.00)	<0.001
**Race [*n* (%)]**				
Mexican American	7,132 (19.44)	5,888 (21.15)	1,244 (14.07)	<0.001
Other Hispanic	2,911 (7.94)	2,371 (8.52)	540 (6.11)	
Non-Hispanic White	16,767 (45.71)	11,522 (41.39)	5,245 (59.31)	
Non-Hispanic Black	7,098 (19.35)	5,732 (20.59)	1,366 (15.45)	
Other Race	2,776 (7.57)	2,328 (8.36)	448 (5.07)	
BMI (kg/m^2^)	27.89 (24.30∼32.22)	27.95 (24.22∼32.56)	27.65 (24.54∼31.35)	<0.001
ALT (U/L)	21.00 (16.00∼28.00)	21.00 (16.00∼30.00)	19.00 (15.00∼24.00)	<0.001
AST (U/L)	23.00 (19.00∼27.00)	22.00 (19.00∼27.00)	23.00 (20.00∼27.00)	<0.001
History of hypertension [*n* (%)]	11,959 (32.75)	6,780 (24.48)	5,179 (58.73)	<0.001
History of diabetes [*n* (%)]	4,015 (10.95)	2,167 (7.79)	1,848 (20.92)	<0.001
Smoking [*n* (%)]	6,299 (17.17)	5,547 (19.92)	752 (8.50)	<0.001
Drinking [*n* (%)]	22,695 (61.87)	17,868 (64.18)	4,827 (54.59)	<0.001
**Components included in BA algorithms**				
Lymphocyte percentage (%)	29.90 (24.50∼35.70)	30.50 (25.30∼36.20)	27.60 (22.20∼33.60)	<0.001
Mean cell volume (fl)	89.80 (86.60∼92.90)	89.40 (86.10∼92.30)	91.40 (88.20∼94.40)	<0.001
Red cell distribution width (%)	12.80 (12.30∼13.50)	12.70 (12.20∼13.40)	13.10 (12.50∼13.90)	<0.001
White blood cell count (1,000 cells/μl)	7.00 (5.70∼8.40)	7.10 (5.80∼8.60)	6.80 (5.70∼8.10)	<0.001
C-reactive protein (mg/dl)	0.21 (0.08∼0.48)	0.20 (0.08∼0.48)	0.23 (0.10∼0.48)	<0.001
Glycated hemoglobin (%)	5.50 (5.20∼5.80)	5.40 (5.10∼5.70)	5.70 (5.40∼6.10)	<0.001
Albumin (g/dl)	4.20 (4.00∼4.50)	4.30 (4.00∼4.50)	4.20 (4.00∼4.40)	<0.001
Systolic blood pressure (mmHg)	121.33 (111.33∼134.67)	118.00 (109.33∼128.67)	135.33 (123.00∼150.67)	<0.001
Alkaline phosphatase (U/L)	69.00 (56.00∼84.00)	68.00 (56.00∼83.00)	71.00 (58.00∼87.00)	<0.001
Blood urea nitrogen (mg/dl)	13.00 (10.00∼16.00)	12.00 (9.00∼15.00)	16.00 (13.00∼20.00)	<0.001
Total cholesterol (mg/dl)	194.00 (168.00∼223.00)	194.00 (169.00∼222.00)	194.00 (166.00∼224.00)	0.082
Glucose (mmol/L)	5.11 (4.72∼5.66)	5.00 (4.61∼5.50)	5.44 (5.00∼6.27)	<0.001
Creatinine (mg/dl)	0.82 (0.70∼1.00)	0.80 (0.70∼0.95)	0.92 (0.80∼1.10)	<0.001
**Calculated biological age**				
KDMAge	41.28 (28.81∼56.34)	35.67 (25.87∼47.70)	61.50 (50.55∼73.77)	<0.001
KDMAge acceleration	−5.86 (−14.86 to ∼3.15)	−4.40 (−12.42 to ∼3.91)	−12.30 (−22.58 to ∼−0.91)	<0.001
Accelerated KDMAge [*n* (%)]	12,060 (32.88)	9,987 (35.87)	2,073 (23.44)	<0.001
PhenoAge	45.01 (30.07∼61.47)	37.63 (26.72∼49.28)	71.51 (65.22∼77.89)	<0.001
PhenoAge acceleration	−3.72 (−6.69 to ∼−0.17)	−3.95 (−6.79 to ∼−0.57)	−2.93 (−6.28 to ∼1.24)	<0.001
Accelerated PhenoAge [*n* (%)]	8,876 (24.20)	6,137 (22.04)	2,739 (30.97)	<0.001

BMI, body mass index; ALT, alanine aminotransferase; AST, aspartate aminotransferase; KDMAge, Klemera–Doubal method age; PhenoAge, phenotypic age; KDMAge acceleration, the residual of the regression of KDMAge based on chronological age; PhenoAge acceleration, the residual of the regression of PhenoAge based on chronological age; Accelerated KDMAge, KDMAge acceleration more than 0; Accelerated PhenoAge, PhenoAge acceleration more than 0.

The median KDMAge for the under-65 and over-65 groups were 35.67 and 61.50 years, respectively, and the median PhenoAge were 37.63 and 71.51 years, respectively. Moreover, both KDMAge acceleration and PhenoAge acceleration were significantly more pronounced in the over-65 group than in the under-65 group (*P* < 0.001). The proportions of accelerated KDMAge and PhenoAge were also significantly higher in the over-65 group (23.44% vs. 35.87% for KDMAge; 30.97% vs. 22.04% for PhenoAge, *P* < 0.001). In addition, plots of biological age vs. chronological age, as shown in Supplementary Figure 1. Compared to the under-65 group, the over-65 group shows a weaker correlation between KDMAge and PhenoAge with chronological age, although the positive correlation is still statistically significant.

In terms of the components included in the BA algorithms, the over-65 group showed significant statistical differences compared to the under-65 group. Specifically, the over-65 group had significantly lower percentages of lymphocytes, white blood cell counts, and albumin levels, while mean cell volume, red cell distribution width, C-reactive protein, glycated hemoglobin, systolic blood pressure, alkaline phosphatase, blood urea nitrogen, blood sugar, and creatinine levels were significantly higher (all *P* values < 0.001). There were no significant differences between the two groups in total cholesterol levels (*P* = 0.082). These results reflect the significant impact of age on these biomarkers.

### 3.2 Chronological age and CRC prevalence

**FIGURE 2:**
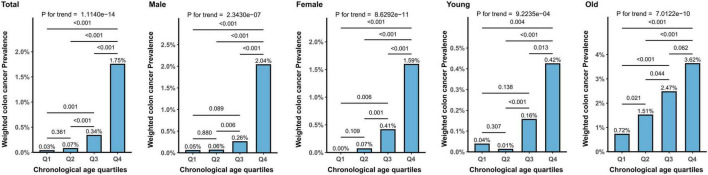
The weighted prevalence of CRC across chronological age quartiles.

Figure 2 presents the distribution of the weighted prevalence of CRC across age quartiles. A significant difference in the prevalence of CRC was observed among the age quartiles in the total population (*P* < 0.001), showing an increasing trend with age (*P* for trend < 0.001). The prevalence rates from Q1 (lowest quartile) to Q4 (highest quartile) were 0.03%, 0.07%, 0.34%, and 1.75%, respectively, with all differences being statistically significant.

In both males and females, the prevalence of CRC significantly increased with age. For males, the quartile prevalences were 0.05%, 0.06%, 0.26%, and 2.04% (*P* for trend < 0.001), and for females, they were 0.00%, 0.07%, 0.41%, and 1.59% (*P* for trend < 0.001). Age group-based analysis showed that in both the younger group (under 65 years) and the older group (65 years and above), the prevalence of CRC significantly increased with age. For the younger group, the quartile prevalences were 0.04%, 0.01%, 0.16%, and 0.42% (*P* for trend < 0.001), and for the older group, they were 0.72%, 1.51%, 2.47%, and 3.62% (*P* for trend < 0.001). Overall, the results indicate a significant increase in the prevalence of CRC in the higher age quartiles, and this trend is consistent across different genders and age groups.

### 3.3 BA measures and CRC prevalence

**FIGURE 3:**
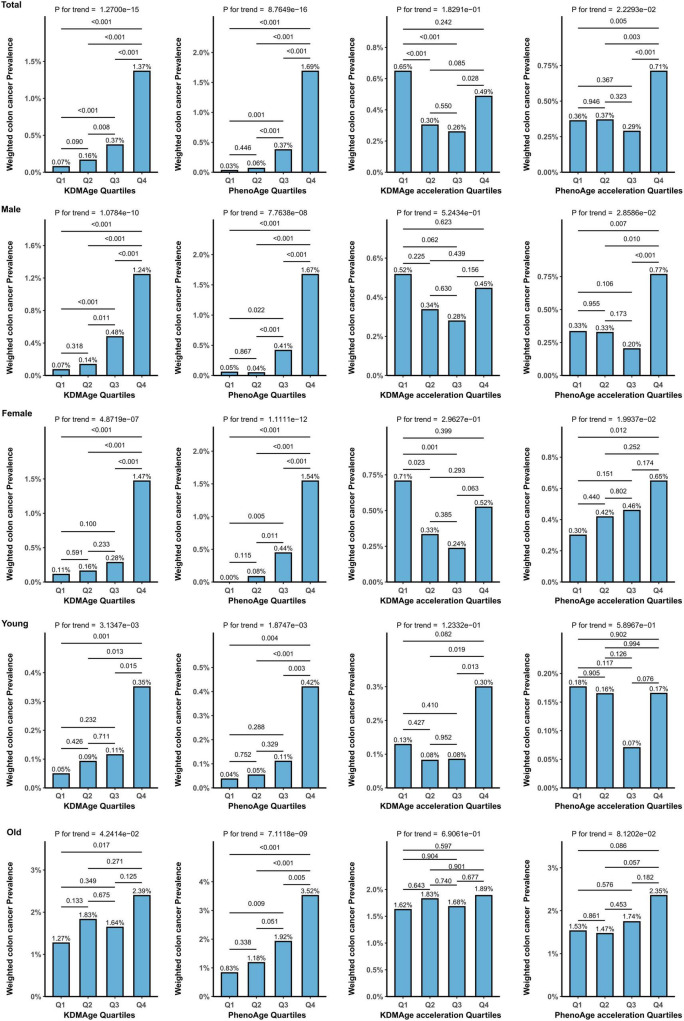
The weighted prevalence of CRC across the quartile of BA measures.

Figure 3 displays the relationship between different BA quartiles and the prevalence of CRC. In the overall population, the weighted prevalence of CRC significantly increased with higher quartiles of both KDMAge (*P* < 0.001) and PhenoAge (*P* < 0.001) with significant trends (all *P* for trend < 0.001). Further analysis examined the relationship between accelerated quartiles of KDMAge and PhenoAge and the prevalence of CRC. Results showed no significant increase in CRC prevalence with higher quartiles of accelerated KDMAge (*P* for trend = 0.183). However, the prevalence rates for accelerated PhenoAge quartiles were 0.36% (Q1), 0.37% (Q2), 0.29% (Q3), and 0.71% (Q4), with a significant trend (*P* for trend = 0.022).

Stratified analysis by gender revealed that both males and females showed significant positive correlations between increasing quartiles of KDMAge and PhenoAge and higher rates of CRC. Additionally, age-stratified analysis indicated that both younger and older groups exhibited significant positive correlations between increasing quartiles of KDMAge and PhenoAge and the prevalence of CRC. Furthermore, accelerated PhenoAge showed a significant positive correlation with CRC prevalence in both males and females, although this trend was not statistically significant in the age-stratified groups. In contrast, accelerated KDMAge showed no significant trend with CRC prevalence in the overall population or any subgroups.

### 3.4 Association of BA measures with CRC prevalence

Table 2 summarizes the associations between BA and the prevalence of CRC under different adjusted models. In the total population, KDMAge was significantly associated with an increased prevalence of CRC, with ORs for model 1, model 2, and model 3 being 2.137 (95% CI: 1.957–2.334, *P* < 0.001), 1.740 (95% CI: 1.552–1.951, *P* < 0.001), and 1.722 (95% CI: 1.532–1.934, *P* < 0.001), respectively. The association with PhenoAge was even more significant, with ORs for model 1, model 2, and model 3 being 4.024 (95% CI: 3.319–4.880, *P* < 0.001), 3.494 (95% CI: 2.823–4.325, *P* < 0.001), and 3.466 (95% CI: 2.813–4.272, *P* < 0.001), respectively. However, accelerated KDMAge did not show a significant association in any models (all *P* > 0.05). In contrast, accelerated PhenoAge significantly associated with increased prevalence of CRC in all models, with ORs for model 1, model 2, and model 3 being 2.173 (95% CI: 1.537–3.071, *P* < 0.001), 1.617 (95% CI: 1.127–2.319, *P* = 0.009), and 1.767 (95% CI: 1.236–2.524, *P* = 0.002), respectively.

**TABLE 2:** The association between biological age and colon cancer risk.

	Model 1		Model 2		Model 3	
	OR (95% CI)	*P*-value	OR (95% CI)	*P*-value	OR (95% CI)	*P*-value
**Total population**						
KDMAge	2.137 (1.957–2.334)	<0.001	1.740 (1.552–1.951)	<0.001	1.722 (1.532–1.934)	<0.001
Accelerated KDMAge	0.978 (0.629–1.521)	0.922	0.819 (0.518–1.294)	0.388	0.847 (0.533–1.344)	0.477
PhenoAge	4.024 (3.319–4.880)	<0.001	3.494 (2.823–4.325)	<0.001	3.466 (2.813–4.272)	<0.001
Accelerated PhenoAge	2.173 (1.537–3.071)	<0.001	1.617 (1.127–2.319)	0.009	1.767 (1.236–2.524)	0.002
**Males**						
KDMAge	2.100 (1.856–2.377)	<0.001	1.795 (1.502–2.146)	<0.001	1.781 (1.487–2.135)	<0.001
Accelerated KDMAge	1.076 (0.622–1.863)	0.792	0.828 (0.477–1.436)	0.499	0.861 (0.495–1.497)	0.593
PhenoAge	3.912 (2.952–5.183)	<0.001	3.615 (2.733–4.780)	<0.001	3.563 (2.699–4.703)	<0.001
Accelerated PhenoAge	2.590 (1.587–4.226)	<0.001	1.751 (1.060–2.891)	0.029	1.934 (1.162–3.220)	0.012
**Females**						
KDMAge	2.169 (1.912–2.461)	<0.001	1.679 (1.426–1.977)	<0.001	1.656 (1.407–1.950)	<0.001
Accelerated KDMAge	0.907 (0.480–1.712)	0.761	0.804 (0.416–1.555)	0.514	0.825 (0.427–1.595)	0.564
PhenoAge	4.106 (3.148–5.355)	<0.001	3.379 (2.414–4.729)	<0.001	3.383 (2.446–4.681)	<0.001
Accelerated PhenoAge	1.850 (1.140–3.002)	0.013	1.572 (0.939–2.631)	0.085	1.704 (1.026–2.831)	0.04
**Age < 65 years**						
KDMAge	1.949 (1.612–2.356)	<0.001	1.651 (1.341–2.033)	<0.001	1.655 (1.334–2.053)	<0.001
Accelerated KDMAge	2.291 (1.012–5.186)	0.047	1.771 (0.763–4.108)	0.181	1.801 (0.770–4.214)	0.173
PhenoAge	2.745 (1.881–4.005)	<0.001	2.248 (1.516–3.332)	<0.001	2.308 (1.516–3.514)	<0.001
Accelerated PhenoAge	1.351 (0.638–2.861)	0.428	0.904 (0.449–1.821)	0.777	0.964 (0.490–1.898)	0.915
**Age ≥ 65 years**						
KDMAge	1.181 (1.010–1.380)	0.037	1.107 (0.929–1.318)	0.253	1.108 (0.932–1.317)	0.244
Accelerated KDMAge	1.062 (0.669–1.687)	0.797	0.931 (0.571–1.517)	0.772	0.937 (0.574–1.528)	0.792
PhenoAge	1.532 (1.342–1.749)	<0.001	1.490 (1.303–1.703)	<0.001	1.497 (1.311–1.710)	<0.001
Accelerated PhenoAge	1.694 (1.170–2.453)	0.006	1.610 (1.106–2.345)	0.013	1.655 (1.143–2.397)	0.008

Model 1: adjusted for age, race and gender; model 2: additionally adjusted for BMI, history of hypertension, and history of diabetes, based on model 1; model 3: further adjusted for smoking and drinking status, based on model 2. Results are expressed as odds ratios (ORs) with 95% confidence intervals (CIs) and *P* values. SD means a standard deviation increase after standardization. KDMAge, Klemera–Doubal method age; PhenoAge, phenotypic age; KDMAge acceleration, the residual of the regression of KDMAge based on chronological age; PhenoAge acceleration, the residual of the regression of PhenoAge based on chronological age; Accelerated KDMAge, KDMAge acceleration more than 0; Accelerated PhenoAge, PhenoAge acceleration more than 0.

In gender-stratified analyses, the results were generally consistent between males and females. Both KDMAge and PhenoAge were significantly associated with the prevalence of CRC, and accelerated PhenoAge significantly associated with increased prevalence of CRC in fully adjusted models (*P* < 0.05), whereas accelerated KDMAge did not show a significant association. Age-stratified analyses revealed that both KDMAge and PhenoAge significantly increased the prevalence of CRC in both the young and old groups. In the young group, neither accelerated KDMAge nor PhenoAge showed significant associations in any model (*P* > 0.05). However, in the old group, accelerated PhenoAge significantly associated with increased prevalence of CRC, with a OR of 1.655 (95% CI: 1.143–2.397, *P* = 0.008) in fully adjusted model. Overall, the results indicate that higher PhenoAge and accelerated PhenoAge significantly associated with increased prevalence of CRC, especially among older adults.

## 4 Discussion

This study utilized the nationally representative NHANES dataset to comprehensively assess the association between BA and the risk of CRC. After accounting for traditional confounders of CRC, we found a significant positive correlation between PhenoAge acceleration and CRC prevalence, especially in the population aged 65 and over. These findings provide a new perspective on the impact of BA on the occurrence of CRC and offer new strategies for early identification of populations at high risk. By measuring biological age, clinicians can assess individualized health risks that may not be visible through traditional medical evaluations. For example, a 50-year-old patient with a biological age closer to 70 might have a higher risk of CRC, and thus could be prioritized for screening, early intervention, or lifestyle modifications. To apply these methods, clinics would need access to tests that measure key biomarkers linked to biological aging (such as albumin, C-reactive protein, blood pressure, cholesterol levels, etc.). These biomarkers would be tested as part of routine check-ups or at-risk screenings.

The incidence of CRC is closely associated with age. Previous research indicates that patients over 75 years old comprise more than 40% of all diagnosed CRC cases, and individuals over 85 years old have more than three times the risk of those aged 60–69 (20, 21). These findings are consistent with our study, where higher age quartiles show higher rates of CRC. Although aging is a significant and unmodifiable risk factor for CRC, it does not necessarily imply a deterioration in population health. As previous research has noted, chronological age does not accurately reflect an individual’s true aging status, nor does it identify those who are genuinely older at the same chronological age (11). In other words, chronological age alone cannot explain the variability in CRC risk among individuals of the same age. Compared to chronological age, BA—calculated based on biomarkers that reflect an individual’s aging state—more accurately represents one’s physiological condition and the risk of age-related diseases and mortality (16). KDMAge and PhenoAge are measures of BA based on a range of clinical markers that broadly reflect the body’s inflammatory status, metabolic function, and the health of the immune system. Markers such as creatinine, C-reactive protein, alkaline phosphatase, and glycated hemoglobin are particularly closely linked to the development of CRC (22–25). For instance, CRP is an inflammatory marker, and elevated levels are often associated with chronic inflammatory responses, which are a key mechanism in the development of CRC (23, 26). Creatinine levels reflect kidney function, and studies suggest that renal dysfunction might increase CRC risk by affecting the clearance of metabolites in the body (22, 26). Alkaline phosphatase is linked to the risk of bone metastasis in CRC, while elevated glycated hemoglobin, an indicator of long-term glucose control, is closely associated with metabolic syndrome and a higher incidence of CRC (24, 27–29). Therefore, calculations of KDMAge and PhenoAge not only predict an individual’s BA but may also provide critical reference for early screening and prevention of CRC.

Our study transcends the limitations of chronological age by utilizing BA, a more reflective indicator of the aging process, to explore the relationship between accelerated aging and the prevalence of CRC. After adjusting for confounders such as chronological age, we found that increases in BA and accelerated biological aging remain significant factors for increased prevalence of CRC. Furthermore, these findings are consistent across different genders. Notably, in the elderly population, higher levels of PhenoAge significantly increased the prevalence of CRC. After comprehensive model adjustments, results showed that each unit increase in PhenoAge among the elderly increased the prevalence of CRC by 76.7%. Even after correcting for actual age and other CRC risk factors, accelerated PhenoAge remained significantly associated with an increased prevalence of CRC. However, this correlation was not observed in the younger population. These findings suggest that accelerated levels of PhenoAge are significant predictors of CRC prevalence in the elderly, emphasizing the need for this population to promptly pay attention to and regularly check CRC-related markers. This also provides a clinically utilizable biological means to identify high-risk individuals among the elderly. Therefore, independent of the increase in chronological age, the intensified degree of biological aging behind accelerated BA becomes a covert factor for CRC risk. This offers new evidence for the unexplained residual risk increase in CRC associated with population aging, and is crucial for the precise identification of early-risk groups for CRC, particularly in the prevention of CRC, especially targeted toward the elderly.

The observed similarities arise among models because both KDMAge and PhenoAge aim to estimate biological aging using overlapping biomarker data. Both methods estimate biological age using clinical biomarkers that reflect aging processes such as inflammation, metabolic function, and immune health. However, KDMAge assumes biological age increases linearly with chronological age, while PhenoAge assumes biological age increases exponentially with mortality risk (16, 17). This fundamental difference means that PhenoAge tends to capture more dramatic deviations from chronological age in older adults, leading to potentially stronger associations with diseases like CRC. PhenoAge includes inflammatory markers (C-reactive protein) and red blood cell indices, which are strongly linked to cancer and other chronic diseases. KDMAge includes more metabolic and organ function markers (systolic blood pressure, blood urea nitrogen, total cholesterol, etc.), which might not capture disease risk as effectively as PhenoAge in some cases. This difference might explain why PhenoAge acceleration rather than KDMAge acceleration was significantly associated with CRC prevalence in this study. Accelerated PhenoAge showed a significant association with CRC, while accelerated KDMAge did not. This could be because PhenoAge acceleration is more sensitive to deviations from normal aging, particularly in populations at risk for cancer. Since PhenoAge explicitly models mortality risk, it may better capture risk factors that contribute to CRC. This study found that accelerated PhenoAge was significantly associated with CRC prevalence only in individuals over 65 years old. This aligns with the idea that PhenoAge is more sensitive to late-life disease processes and may be better at detecting aging-related deterioration that leads to CRC.

This study represents the first exploration of the relationship between BA and CRC based on U.S. adult population. Moreover, this study adjusted for common risk factors when calculating the impact of BA acceleration on CRC prevalence, with particular controls for physiological age and gender in detailed subgroup analyses. However, the study has several limitations. First, the cross-sectional design limits the ability to establish causality. We cannot definitively determine whether accelerated biological aging increases the risk of CRC, or if the presence of CRC accelerates biological aging. Alternative explanations like reverse causality and the possibility that biological aging might be a marker of broader health declines, rather than a direct cause of CRC. Second, some variables were obtained through surveys and self-reported questionnaires, making them susceptible to bias. Some individuals may not accurately report their history of CRC, especially in the case of undiagnosed or asymptomatic cases, leading to potential misclassification. Self-reports on lifestyle factors, such as diet, physical activity, and smoking status, may not accurately reflect participants’ true behaviors. Third, while our study adjusted for several key covariates, there may still be unmeasured confounders that could influence the observed associations, such as genetic predisposition, environmental exposures, healthcare access, and other unmeasured variables. Forth, years since diagnosis of CRC cannot be obtained. Lastly, this study did not explore the relationship between BA and CRC prevalence across different racial groups. In the future, KDMAge and PhenoAge algorithms would need to be incorporated into clinical decision-making tools. This could involve software platforms or clinical calculators that take a patient’s test results and compute their biological age, comparing it to their chronological age. This would allow healthcare providers to easily assess whether a patient has accelerated biological aging.

## 5 Conclusion

In conclusion, accelerated biological aging is associated with the increased prevalence of CRC, particularly among individuals aged 65 and older. These findings provide a new strategy for the early identification of at-risk populations.

## Data Availability

Publicly available datasets were analyzed in this study. This data can be found here: https://www.cdc.gov/nchs/nhanes/.
