# Supplementary figures and images for: Association between accelerated biological aging and colorectal cancer: a cross-sectional study
Source: Front Med (Lausanne). 2025 Feb 21;12:1533507. doi: 10.3389/fmed.2025.1533507 (PMC11885229; doi:10.3389/fmed.2025.1533507)

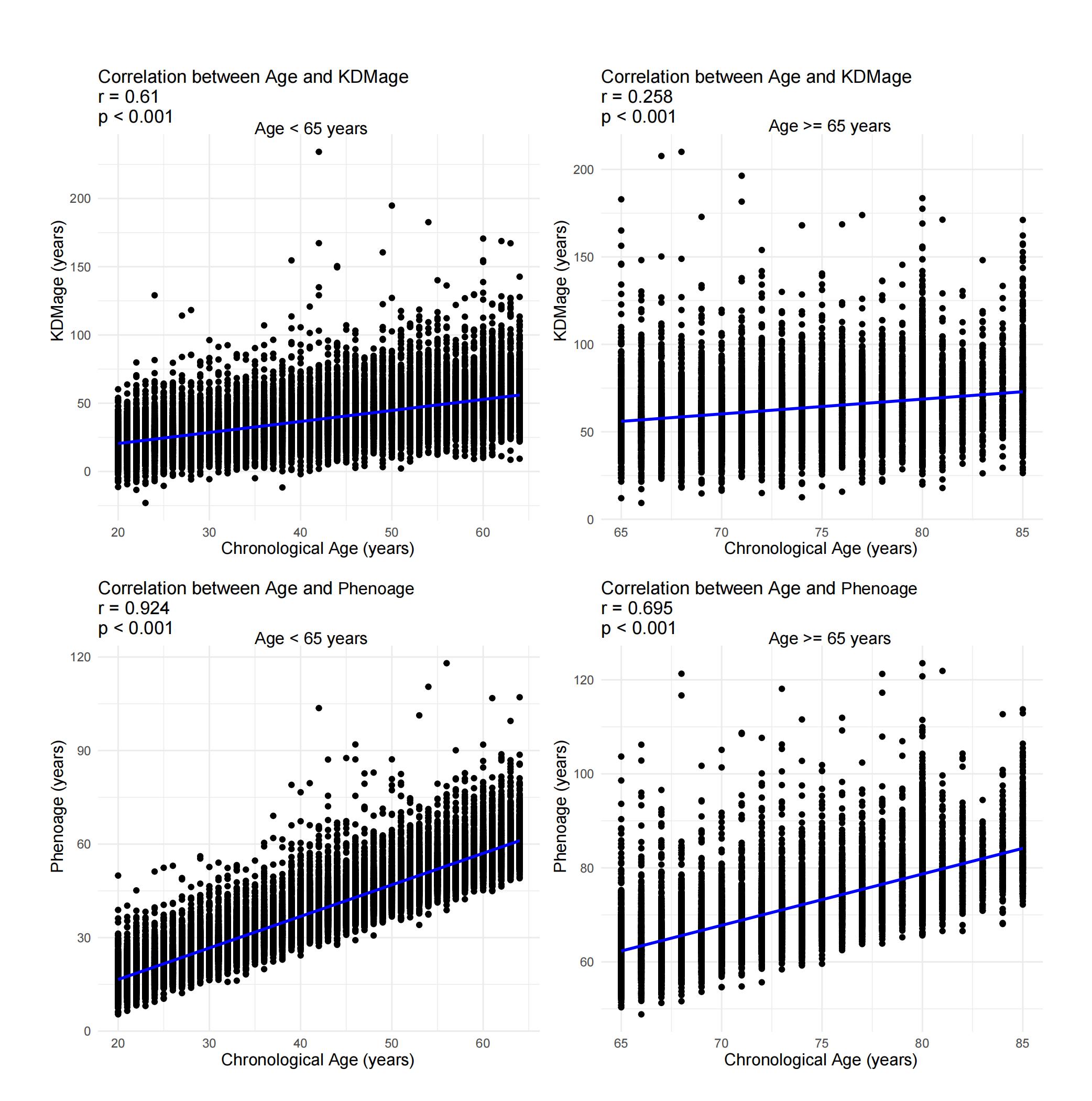

Supplement: Supplementary file 1 [file Image_1.jpeg]
