# Supplementary material for: Association between accelerated biological aging and colorectal cancer: a cross-sectional study
Source: Front Med (Lausanne). 2025 Feb 21;12:1533507. doi: 10.3389/fmed.2025.1533507 (PMC11885229; doi:10.3389/fmed.2025.1533507)
Supplement: Supplementary file 2 [file Table_1.docx]

Supplementary Material

included in the file and not in the manuscript, and that the style conforms to the rest of the article.

# Supplementary Tables

Supplementary table 1. Weighted basic characteristics of the study population

|  | Overall | Age ＜ 65 years | Age ≥ 65 years | P value |
| --- | --- | --- | --- | --- |
| **N** | 155415659.7 | 128890770.9 | 26524888.87 |  |
| **Age (years)** | 45.00 (33.00~59.00) | 41.00 (31.00~51.00) | 73.00 (68.00~79.00) | <0.001 |
| **Race (n %)** |  |  |  |  |
| Mexican American | 12755806.90 (8.21) | 11815919.84 (9.17) | 939887.06 (3.54) | <0.001 |
| Other Hispanic | 8532194.38 (5.49) | 7678126.64 (5.96) | 854067.74 (3.22) |  |
| Non-Hispanic White | 107581909.31 (69.22) | 85914502.91 (66.66) | 21667406.40 (81.69) |  |
| Non-Hispanic Black | 16217410.91 (10.43) | 14309512.04 (11.10) | 1907898.87 (7.19) |  |
| Other Race | 10328338.24 (6.65) | 9172709.45 (7.12) | 1155628.79 (4.36) |  |
| **BMI (kg/m^2^)** | 27.60 (24.03~32.02) | 27.58 (23.93~32.18) | 27.62 (24.57~31.48) | 0.452 |
| **ALT (U/L)** | 21.00 (16.00~29.00) | 22.00 (16.00~30.00) | 19.00 (15.00~25.00) | <0.001 |
| **AST (U/L)** | 22.00 (19.00~27.00) | 22.00 (19.00~27.00) | 23.00 (20.00~27.00) | <0.001 |
| **History of hypertension (n %)** | 44424533.50 (28.68) | 29286689.77 (22.81) | 15137843.73 (57.19) | <0.001 |
| **History of diabetes (n %)** | 12371331.75 (7.96) | 7562447.22 (5.87) | 4808884.53 (18.15) | <0.001 |
| **Smoking (n %)** | 28234001.27 (18.17) | 26220147.63 (20.34) | 2013853.64 (7.59) | <0.001 |
| **Drinking (n %)** | 103827452.05 (66.81) | 89009091.22 (69.06) | 14818360.83 (55.87) | <0.001 |
| **Components included in BA algorithms** |  |  |  |  |
| lymphocyte percentage (%) | 29.80 (24.60~35.10) | 30.20 (25.20~35.50) | 27.30 (22.10~33.10) | <0.001 |
| Mean cell volume (fL) | 90.00 (86.90~92.90) | 89.70 (86.70~92.60) | 91.60 (88.60~94.40) | <0.001 |
| Red cell distribution width (%) | 12.70 (12.20~13.40) | 12.60 (12.20~13.30) | 13.10 (12.50~13.80) | <0.001 |
| White blood cell count (1000 cells/uL) | 7.00 (5.80~8.40) | 7.10 (5.80~8.50) | 6.80 (5.70~8.10) | <0.001 |
| C-reactive protein (mg/dL) | 0.18 (0.07~0.43) | 0.18 (0.07~0.43) | 0.21 (0.10~0.45) | <0.001 |
| Glycated hemoglobin (%) | 5.40 (5.10~5.70) | 5.30 (5.10~5.60) | 5.70 (5.40~6.00) | <0.001 |
| Albumin (g/dL) | 4.30 (4.10~4.50) | 4.30 (4.10~4.50) | 4.20 (4.00~4.40) | <0.001 |
| Systolic blood pressure (mmHg) | 120.00 (110.67~131.33) | 118.00 (109.33~128.00) | 134.00 (121.33~148.67) | <0.001 |
| Alkaline phosphatase (U/L) | 67.00 (55.00~81.00) | 66.00 (54.00~81.00) | 70.00 (57.00~86.00) | <0.001 |
| Blood urea nitrogen (mg/dL) | 13.00 (10.00~16.00) | 12.00 (10.00~15.00) | 16.00 (13.00~20.00) | <0.001 |
| Total cholesterol (mg/dL) | 194.00 (169.00~222.00) | 194.00 (169.00~222.00) | 196.00 (168.00~226.00) | 0.098 |
| Glucose (mmol/L) | 5.05 (4.66~5.55) | 5.00 (4.61~5.44) | 5.38 (4.94~6.11) | <0.001 |
| Creatinine (mg/dL) | 0.82 (0.70~1.00) | 0.81 (0.70~0.97) | 0.90 (0.79~1.10) | <0.001 |
| **Calculated biological age** |  |  |  |  |
| KDMAge | 38.39 (27.44~51.82) | 34.90 (25.59~46.00) | 60.11 (49.53~71.43) | <0.001 |
| KDMAge acceleration | -6.13 (-14.66~2.35) | -5.12 (-13.03~2.95) | -13.14 (-22.71~-2.27) | <0.001 |
| Accelerated KDMAge (n %) | 48516235.79 (31.22) | 43038241.36 (33.39) | 5477994.42 (20.65) | <0.001 |
| PhenoAge | 41.62 (28.84~55.71) | 37.28 (26.60~47.72) | 70.24 (64.33~76.58) | <0.001 |
| PhenoAge acceleration | -4.12 (-6.90~-0.88) | -4.29 (-6.98~-1.17) | -3.27 (-6.41~0.65) | <0.001 |
| Accelerated PhenoAge (n %) | 31798223.74 (20.46) | 24211937.18 (18.78) | 7586286.56 (28.60) | <0.001 |

BMI, body mass index; ALT, alanine aminotransferase; AST, aspartate aminotransferase; KDMAge, Klemera-Doubal method age; PhenoAge, phenotypic age;KDMAge acceleration, the residual of the regression of KDMAge based on chronological age; PhenoAge acceleration, the residual of the regression of PhenoAge based on chronological age; Accelerated KDMAge, KDMAge acceleration more than 0; Accelerated PhenoAge, PhenoAge acceleration more than 0.
